# 517 The Hyperinflammatory Response Is Not Further Exacerbated Using Plasma During Resuscitation

**DOI:** 10.1093/jbcr/iraf019.146

**Published:** 2025-04-01

**Authors:** Andrew Bieterman, Amanda Soo Ping Chow, Tuan Le, Melissa McLawhorn, Shawn Tejiram, Taryn Travis, Lauren Moffatt, Jeffrey Shupp

**Affiliations:** The Burn Center at MedStar Washington Hospital Center; The Burn Center at MedStar Washington Hospital Center; MedStar Health Research Institute; MedStar Health Research Institute; MedStar Washington Hospital Center; MedStar Washington Hospital Center; MedStar Health Research Institute; MedStar Washington Hospital Center

## Abstract

**Introduction:**

Burn injuries induce a hypermetabolic and systemic inflammatory response that is difficult to mitigate. The post-burn inflammatory response is mediated by cytokines such as tumor necrosis factor alpha (TNF-α), (interleukin-6) IL-6, and interleukin-8 (IL-8). Advances in modern burn resuscitation have dramatically decreased the mortality associated with burn shock. However, prolonged inflammatory response following severe burns can be detrimental, leading to increased susceptibility to infections, multi-organ failure and death. Recently, there has been a shift towards the earlier use of colloids, such as albumin and fresh frozen plasma (FFP), to reduce the volume of crystalloids required to maintain end-organ perfusion during resuscitation. Previous literature has suggested that FFP use may increase the risk of exacerbating the host systemic immune response. The effect of FFP transfusion on cytokine host response in burn patients is currently unknown. In this study, we investigated the effects of FFP administration on the biomarkers of inflammation during resuscitation.

**Methods:**

A prospective study was performed on burn patients with >20% TBSA burns who underwent plasma inclusive burn resuscitation. Blood samples were collected within 4 hours of admission, immediately prior to and after administration of the first unit of FFP and after resuscitation was complete. Serum concentrations of IL-6, soluble IL-6 receptor (sIL-6R), TNF-α, TNF-α receptor (TNF-αR) were quantified using enzyme-linked immunosorbent assay (ELISA). Statistical analysis was performed using Friedman’s multiple comparison test to compare the serum concentrations prior to and after FFP administration.

**Results:**

Twenty-nine patients were included in the analysis. The patients were predominantly male (75.9%) with a median age of 46 years and a median TBSA burn of 34%. The overall mortality was 27.6%. At baseline, IL-6 and TNF-α were elevated compared to normal reference ranges. IL-6 levels were higher after resuscitation [708 (374-1736)], than baseline [133 (66-270); p< 0.0001], pre-plasma [175 (90-340); p< 0.0001] and after one unit [152 (93-405); p=0.0008]. Similarly, TNF-α levels were higher post-resuscitation [14 (12-17)], compared to baseline [10 (9-13); p< 0.0001], pre-plasma [12 (9-14); p< 0.0001] and post-unit [12 (9-14); p=0.0001]. Levels of IL-6, TNF-α and their receptors did not differ pre-plasma and post-unit.

**Conclusions:**

The administration of FFP did not worsen the systemic inflammatory response seen in severe burn injuries.

**Applicability of Research to Practice:**

Concerns regarding exacerbating the systemic inflammatory response should not preclude the use of FFP as an adjunct for resuscitation based on this data. Additional research should be directed towards comparing the immune response of other colloids that can be used as resuscitation adjuncts.

**Funding for the Study:**

N/A

